# The “*Journal of Functional Morphology and Kinesiology*” Journal Club Series: Resistance Training

**DOI:** 10.3390/jfmk5020025

**Published:** 2020-04-02

**Authors:** Antonio Paoli, Tatiana Moro, Silvio Lorenzetti, Jan Seiler, Fabian Lüthy, Micah Gross, Federico Roggio, Helmi Chaabene, Giuseppe Musumeci

**Affiliations:** 1 Department of Biomedical Sciences, University of Padova, 35131 Padova, Italy; 2 Swiss Federal Institute of Sport Magglingen (SFISM), 2532 Magglingen, Switzerland; 3 Department of Biomedical and Biotechnological Sciences, Anatomy, Histology and Movement Sciences Section, School of Medicine, University of Catania, Via S. Sofia 87, 95123 Catania, Italy; 4 Division of Training and Movement Sciences, University of Potsdam, Am Neuen Palais 10, 14469 Potsdam, Germany; 5 Research Center on Motor Activities (CRAM), University of Catania, 95123 Catania, Italy; 6 Department of Biology, Sbarro Institute for Cancer Research and Molecular Medicine, College of Science and Technology, Temple University, Philadelphia, PA 19122, USA

## Abstract

We are glad to introduce the Second Journal Club of Volume Five, Second Issue. This edition is focused on relevant studies published in the last few years in the field of resistance training, chosen by our Editorial Board members and their colleagues. We hope to stimulate your curiosity in this field and to share with you the passion for the sport, seen also from the scientific point of view. The Editorial Board members wish you an inspiring lecture.

## 1. Introduction

Resistance training, also known as strength training, is a form of exercise that improves muscular strength and endurance [1,2,3]. During a resistance training workout, limbs move against resistance provided by your body weight, gravity, bands, weighted bars, or dumbbells. Some exercise machines can also be used for resistance training. Any exercise where the body moves against resistance can be considered resistance or strength training [1,2,3]. The definition of resistance in this form of training is simple as well. Resistance is any force that makes the movement harder to perform. Resistance can be provided simply by moving your body against gravity; by adding weighted dumbbells or using machines at the gym; or by using equipment such as weighted bars, bands, or kettlebells [1,2,3]. This form of training can also be called weight lifting or weight training. The benefits of resistance training are significant for skeletal muscle mass mutation [4,5,6]. Skeletal muscle is a particular tissue that modifies its general functional ability in reaction to varied stimuli [4,5,6]. Strength improvement, as well as increases in muscle cross-sectional area and contractile capacity, noted during the initial phase of training would result from neuromuscular activation in combination with trophic factors [4,5,6]. In order to stimulate further adaptation toward specific training goals, progressive resistance training (RT) protocols are necessary [7]. The optimal characteristics of strength-specific programs include the use of concentric (CON), eccentric (ECC), and isometric muscle actions, and the performance of bilateral and unilateral single- and multiple-joint exercises [7]. Strength programs sequence exercises to optimize the preservation of exercise intensity (large before small muscle group exercises, multiple-joint exercises before single-joint exercises, and higher-intensity before lower-intensity exercises) [7]. These exercises should be applied in adapted contexts and should be contingent upon an individual’s target goals, physical capacity, and training status.

## 2. Biomechanical Analytics of Resistance Training Exercises

### Highlight by Micah Gross and Silvio Lorenzetti

Recently, technologies such as motion capture, force plates, electromyography (EMG), and computer modelling have been used to assess the impact of resistance training on the human body, and to identify differences between exercises and workout configurations. Typically, effects on the kinematic and kinetic characteristics of movements—or on muscle activation patterns—stand in focus, because a good understanding of these facilitates structuring workouts so as to elicit a desired stimulus and achieve particular goals. With velocity-based training methods gaining popularity in recent years, movement velocity has arguably received the most attention among the kinematic variables of strength training exercises. For example, Weakley et al. [8] assessed the effects of three superset configurations on the kinetics and kinematics of the barbell bench press. These authors advised the use of agonist-antagonist supersets, because this configuration allows subjects to best maintain bar velocity and power across the course of the workout. For similar reasons, Wetmore et al. [9] deemed cluster sets of heavy back squats superior to traditional set configurations; they showed in their study that peak velocity and power, as well as the early attainment of these, was better maintained throughout the workout using cluster sets. Further, Morales-Artacho et al. [10] reported similar advantages of set clustering for ballistic squat jumps. Of interest to novice lifters or in rehabilitation situations, kinetic variables such as forces and torques acting on certain joints, as well the interplay of co-activated muscles, have been investigated, so as to determine the safest ways to achieve adequate strength training stimuli. Comparing unilateral and bilateral squats with the same external load per leg, Eliassen et al. [11] found greater muscle force and higher barbell velocity in the unilateral squats, and therefore recommended these for subjects with low back pain and after ACL ruptures. Elsewhere, Mausehund et al. [12] differentiated three similar unilateral lower-limb squat variants in terms of external load and muscle activation, and accordingly recommended single leg squats for alleviating spinal loading, and split squats with the rear foot elevated for ACL patients, because the greater hamstring co-activation in this exercise helps to stabilize the knee joint. Additionally, studies have investigated the effects of assisting devices while performing strength exercises. Using musculo-skeletal modelling techniques, Sinclair et al. [13] analyzed the acute effects of knee wraps on the kinetics, kinematics, and muscle forces during back squats. The knee wraps reduced muscle forces at a given load, and were suspected to therefore diminish the muscle development stimulus. Elsewhere, the proposed benefits of heel wedges while performing barbell back squats have been investigated. Having used motion capture and EMG to assess trunk and pelvis biomechanics, the studies of both Lee et al. [14] and Charlton et al. [15], performed on recreational and resistance-trained subjects, respectively, agree that heel wedges do not facilitate a neutral spine and are therefore unlikely to prevent back injuries while squatting.

## 3. Velocity-Based Monitoring of Resistance Training

### Highlight by Jan Seiler, Fabian Lüthy, and Micah Gross

In many sports, developing high muscular power is considered one of the most important performance-determining factors. Power at a given resistance is highly dependent on movement velocity [16], which is an indirect indicator of muscle contraction velocity, and thus of the intensity of a muscle contraction. Hence, measurements of velocity during training have been deemed a practical means of monitoring loading intensity [17], in addition to external load and volume. In recent years, the application of this principle, which has been coined velocity-based training (VBT), has received increasing attention from researchers. Moreover, the popularity of VBT has increased considerably among practitioners, thanks in large part to the introduction of numerous commercial devices for tracking velocity, which include linear position transducers, accelerometers, and video-based smartphone applications [18,19,20]. Two main characteristics of VBT which differ from traditional strength training methods are (1) the use of velocity zones, rather than percentages of 1-RM, for determining resistance, and (2) the use of velocity thresholds, rather than failure or a predetermined number of repetitions, for terminating sets. The first principle intends to prescribe loads based on the individual load-velocity regression and adjust for day-to-day fluctuations in ability [17]. The second principle aims to ensure only high-quality repetitions and to minimize unnecessary fatigue and metabolic stress accumulated in repetitions outside the intended velocity zone [21,22]. Banyard et al. [23] provided evidence for the claimed benefits of VBT over traditional methods. They showed that, over the course of a training session, prescribed movement velocity was better maintained when load was adjusted between sets based on velocity measurements. Moreover, when sets were terminated based on velocity thresholds rather than a predetermined number of reps, subjects completed either fewer sets for the same number of total reps (which was usually more time efficient) or fewer reps in the same number of sets. The generally higher movement velocities with VBT, and—especially—the case where fewer reps per set were completed, corresponded to overall reduced time under tension, which the authors also purported as a benefit of VBT, since mechanical stress is kept to a minimum. Having applied similar VBT principles over a short training phase, the study of Dorrell et al. [24] provides further support for the use of velocity zones rather than traditional percentages of 1-RM for dictating loads. These authors reported similar or greater training adaptations with reduced training volume for VBT. They concluded that monitoring velocity might better assure that day-to-day training corresponds to the prescribed intensity, while also indicating and compensating for subjects’ current state of fatigue [24]. Terminating sets based on a velocity threshold rather than training to or near to failure is arguably the largest difference between VBT and traditional methods. However, an increasing number of investigations appear to indicate that training to muscular failure may not be necessary to produce increments in muscle strength and that the continuous use of that strategy results in a significant decrement in the rate of force development [25,26]. Furthermore, higher thresholds, which terminate sets even earlier, could be preferable to more lenient thresholds. Indeed, Rodriguez-Rosell et al. showed that when reductions in velocity of only 10% were tolerated before terminating sets, similar or greater changes in strength, muscle endurance, jump performance, and sprint performance occurred with less than half as many repetitions, compared with using 30% velocity loss per set [27]. On a larger scale than individual sessions, fatigue monitoring through the use of movement velocity could help to optimize the periodization and planning of subsequent strength training sessions. In this context, Vernon et al. highlighted that movement velocity at loads ≥ 60% 1-RM may be compromised to a stronger degree than either 1-RM itself or countermovement jump performance in the 24–72 h following a strength session [28]. An additional, psychological advantage of device-assisted VBT is the immediate biofeedback. Nagata et al. [29] showed that a four-week training intervention with immediate numerical velocity feedback increased jump squat performance more compared to training with video-feedback or no feedback. The increased performance was also better sustained 10 days post-intervention [29]. Interestingly, Hirsch et al. showed that oral instructions to lift the bar as fast as possible were not as effective as a numerical velocity target for attaining a high movement velocity in male powerlifters [30]. Despite the apparent benefits of VBT with immediate feedback, the financial investment for a feedback device remains a barrier for some strength and conditioning professionals or athletes. In such cases, it is advisable to err on the side of smaller percentage losses in velocity, which, as recently mentioned by Jukic et al. [31], is well facilitated by employing cluster sets in the absence of any measuring device. So far, research on VBT has either standardized or disregarded the influence of preceding eccentric movements on concentric velocity. In practical settings, however, stretch-shortening cycles of the muscle-tendon unit affect concentric movements strongly. Furthermore, different eccentric speeds can induce different adaptive responses following resistance training [32,33]. Eccentric training and eccentric rates of force development sparked the interest of certain researchers recently [34]. Thus, future research should address the role of eccentric actions on training quality, fatigue, and training effectiveness within the context of VBT.

## 4. Resistance Training for Adults with Musculoskeletal Deficits

### Highlight by Federico Roggio and Giuseppe Musumeci

Injuries to the musculoskeletal system (e.g., lower-extremity injuries, osteoarthritis, etc.) can result in a series of deficits that can lead to reduced long-term health-related quality of life such as impaired gait speed, static and dynamic balance, and stair climbing, and an increased fall risk [35,36,37]. An abnormal gait pattern and a reduced biomechanical symmetry can be linked to a weakness of leg muscles [38,39]. Specifically after the age of 50, muscle strength decreases approximately 15% every 10 years; meanwhile, muscle mass decreases approximately 2% every year [40]. During the past decade, many studies have evaluated the effects of applying resistive loads just in a walking task, and the results indicate that muscles and joints increase their activation, while the metabolic cost increases [41,42,43]. There is strong evidence to suggest that resistance training is an effective method of increasing strength in older adults [44,45]. In order to attenuate the effects of sarcopenia, the recommendation is to perform resistance training 2–3 days per week. There does not exist a definitive gold standard prescription for resistance training exercise because most prescriptions focus on applying a resistive load, which may lead to a training session with low results. In order to reach patient-specific needs, Washabaugh E.P. et al. presented a biomechanical simulation to evaluate the effects of different modes of applying a resistance training during walking [46]. Different methods led to different results, such as quadricep activation rather than hamstring activation. They evaluated five different ways to apply a resistive load, and the results show that (1) a weight attached at the ankle primarily increases resistance to the hamstring muscles; (2) an elastic band attached at the ankle primarily increases resistance to the quadriceps muscles; (3) a viscous device attached to the hip and knee provides resistance to the hamstrings over the stance phase, and to the quadriceps during mid and late swing; (4) a weight attached at the pelvis doesn’t provide an effective improvement; and (5) a constant backwards pulling force at the pelvis increases both quadricep and hamstring activation [47,48]. Thus, when resistance training is used, the correct application method must be selected to ensure improvement according to patient-specific needs.

## 5. The Role of Capillarization on Skeletal Muscle Adaptation to Resistance Training

### Highlight by Antonio Paoli and Tatiana Moro

Resistance training (RT) is the most efficient strategy to promote muscle hypertrophy and strength. Recently, muscle perfusion and capillary density have emerged as crucial predictors of hypertrophic capacity following RT [49,50], both in young and older individuals. Capillaries have the important function of delivering oxygen, nutrients, and hormones in all tissues. Adequate skeletal muscle capillarization can enhance muscle protein synthesis by ensuring the transport of amino acids and growth factors to muscle fibers. In particular, the delivery of systemic signals, such as insulin-like growth factor-1 (IGF-1), hepatocyte growth factor (HGF), interleukin 6 (IL-6), and myostatin, seems to regulate satellite cell (SC) function, supporting muscle repair and/or remodelling/adaptation [51,52]. Nederveen at al. [53] showed that the distance between capillaries and SCs is greater in older adults compared with younger people. They also observed that after a single bout of RT, active SCs move closer to the nearest capillary whilst quiescent SCs remain at a further distance. This finding suggests that local perfusion supports muscle growth also by promoting SC activity, and may help to explain the different anabolic response to RT typical of aging. Timmerman et al. demonstrated that a moderate acute increase in physical activity can improve nutritive blood flow and stimulate the anabolic response to nutrient intake [54]. We have recently showed that 12 weeks of progressive RT can increase muscle strength by 16%, lean mass by 2%, and muscle cross-sectional area by 27% in healthy older adults [49,50,51,52,53,54]. However, our results suggest that the hypertrophic response to RT is linked to the basal level of capillarization. It seems indeed that the early adaptive cellular response to RT is to increase the density of the microvascular bed and the surface exchange area, which can promote the diffusion of nutrients to the tissue. Only when muscle fibers have adequate perfusion can the rate of protein synthesis increase and lead to muscle growth [49]. These findings lead to the hypothesis that a muscle capillarization threshold may be necessary to promote the hypertrophic adaptation to RT.

## 6. What Makes Eccentric More Effective than Concentric and Isometric Muscle Actions?

### Highlight by Helmi Chaabene

There are three categories of muscle action. These are isometric, concentric, and eccentric. While concentric and isometric muscle actions are well investigated, eccentric muscle actions are still understudied in the literature. Unlike the concentric and isometric actions, eccentric actions do not fit well into the well-known cross-bridge theory [55]. The winding filament theory has been proposed [56] as an alternative underlying mechanism of eccentric muscle actions. This has advanced our understanding as to the specific properties of eccentric muscle actions compared with isometric and concentric actions. In the context of the winding theory, it has been suggested that during active lengthening contractions (i.e., eccentric actions), the giant protein titin appears to actively participate in the generation of force by increasing its stiffness when winded up onto actin [57]. Titin is activated by Ca^2+^ release and winds upon the thin filament by the cross-bridges, which also translate as they rotate the thin filament [58].

There is evidence that fundamental differences exist between eccentric and concentric/isometric muscle actions from metabolic and neural perspectives. In terms of metabolic demands, it has been demonstrated that eccentric muscle actions are less taxing and require less energy per unit of work [59,60]. For neural drive, eccentric and concentric muscle actions display considerable differences [61]. Lower electromyography activity during eccentric compared with concentric muscle actions has been reported [62]. This difference seems to be mainly due to a lower number of active motor units in addition to lower discharge rates during eccentric compared with concentric actions [61,63]. Despite the lower metabolic cost and lower neural drive during eccentric actions, higher force production (20–60%) compared with isometric and concentric actions can be observed [64,65]. This makes eccentric actions distinctly more effective and efficient than concentric/isometric actions.

Further eccentric exercises generate a greater hypertrophic response compared with isometric and concentric training [66,67]. This is substantiated by the higher anabolic signalling as reflected by a greater satellite cell activation following eccentric compared with concentric muscle actions [68]. Additionally, eccentric exercises result in greater force compared with isometric and concentric training [66]. Moreover, eccentric exercises have the potential to improve muscle power [69,70]. Overall, there is evidence to favor eccentric training over concentric/isometric training to improve muscle mass, strength, and power.
